# Habitual Coffee Consumption and Systemic Health Outcomes: A Comprehensive Review

**DOI:** 10.7759/cureus.99094

**Published:** 2025-12-13

**Authors:** Eliza Kwapien, Mateusz Piszka, Filip Czarnecki, Marceli Mesyasz, Aleksandra Owczarska, Hubert Dacyl, Jan Banach, Wiktoria Z Kasprzyk, Jan K Luczak, Jakub Bartkowski

**Affiliations:** 1 Medicine, Wojewódzki Szpital Specjalistyczny nr 5 im. św. Barbary, Sosnowiec, POL; 2 Medicine, Szpital Miejski w Siemianowicach Śląskich, Siemianowice Śląskie, POL; 3 Medicine, Zagłębiowskie Centrum Onkologii Szpital Specjalistyczny im. Sz. Starkiewicza w Dąbrowie Górniczej, Katowice, POL; 4 Medicine, Szpital Powiatowy w Limanowej Imienia Miłosierdzia Bożego, Limanowa, POL; 5 Faculty of Medicine, Medical University of Warsaw, Warsaw, POL; 6 Medicine, Miejskie Zakłady Opieki Zdrowotnej w Żorach, Żory, POL

**Keywords:** alzheimer's disease, cardiovascular prevention, coffee consumption, diabetes mellitus type 2, liver cirrhosis, oncology, preventive nephrology

## Abstract

This review analyzes extensive scientific data linking habitual coffee consumption to a wide range of physiological outcomes and divides them into cardiovascular, metabolic, and organ-specific effects. Unlike previous assumptions, moderate coffee intake positively affects many aspects of the health of the adult population. We can observe beneficial effects that translate into patients' longevity as well as reduced risks of chronic diseases. After analyzing the studies, the most significant protective associations were identified in the cardiovascular system, where moderate consumption (three to four cups daily, corresponding to approximately 450-600 mL of coffee) correlates with reduced all-cause and cardiovascular mortality, and a lower risk of heart failure. Many studies have highlighted the chemo-preventive potential of coffee constituents, indicating a reduced risk of specific malignancies, including liver and prostate cancer. The health consequences that manifest themselves clinically also include improved metabolic homeostasis, specifically a lower incidence of type 2 diabetes mellitus and liver fibrosis. This impact also extends to the central nervous system, where there is a clear correlation between caffeine intake and neuroprotection against neurodegenerative disorders such as Alzheimer’s and Parkinson’s disease. Evidence indicates a "J-shaped" relationship in many cases, suggesting that moderate intake is optimal. Conversely, supraphysiological consumption can induce transient hemodynamic disturbances, and unfiltered preparations may negatively impact lipid profiles due to higher diterpene content. Furthermore, evidence supports a linear dose-response relationship between caffeine intake and adverse pregnancy outcomes, specifically pregnancy loss, highlighting the need for strict caution in prenatal care. Due to the proven bioactive properties of compounds like chlorogenic acid and caffeine, updated public health perspectives are needed to recognize coffee not merely as a stimulant, but as a functional dietary component that promotes a healthy lifestyle, which will translate into the reduced burden of non-communicable diseases in the future.

## Introduction and background

In recent years, coffee consumption has become an integral part of the modern diet, and exposure to its bioactive compounds constitutes a significant contributor to the polyphenol content within the European diet [1]. While this beverage acts primarily as a psychoactive agent providing improved alertness and cognitive performance [2], there is growing evidence linking habitual coffee intake to positive health effects and reduced all-cause mortality [3,4]. The consumption of coffee has been linked to a broad spectrum of protective outcomes, extending beyond its traditional role to include cardiovascular, metabolic, and oncological domains [5,6]. This review synthesizes the current scientific understanding of the relationship between habitual coffee consumption and human health. We explore key areas such as the association with major lifestyle diseases like cardiovascular mortality and type 2 diabetes mellitus, as well as the effects on organ-specific health including liver and renal function [7-9]. Furthermore, we delve into the complex but increasingly critical areas of neuroprotection, including the mitigation of neurodegenerative disorders, and the potential chemo-preventive properties against various malignancies [10-12]. By examining these multifaceted relationships, this paper aims to provide a comprehensive overview for clinicians and public health professionals, highlighting the need for updated perspectives and informed guidelines to acknowledge the beneficial effects of moderate coffee consumption and promote a healthier lifestyle for the general population. Finally, remaining controversies and potential adverse effects are addressed, particularly regarding pregnancy outcomes and cardiovascular risks, emphasizing that dosage constitutes a critical factor. A standard cup of coffee contains 150 mL of liquid [1].

## Review

Methodology

To provide a comprehensive overview of the relationship between coffee and human health, we conducted a targeted literature search within the PubMed database, encompassing publications up to 2025. The screening process relied on specific keywords and Medical Subject Headings (MeSH) such as "habitual coffee consumption", "caffeine", "cardiovascular health", "metabolic homeostasis", "neuroprotection", and "chemo-prevention". We restricted our selection to English-language articles focusing on adult populations, with a strong prioritization of high-level evidence. Consequently, the core of this synthesis is derived from systematic reviews and dose-response meta-analyses, supplemented by large prospective cohort studies where necessary to ensure a thorough representation of the field. As this work is structured as a narrative review rather than a systematic meta-analysis, we did not apply formal risk-of-bias assessment tools; instead, studies were critically evaluated for their methodological clarity and relevance to the specific health domains discussed. Furthermore, no new statistical computations were performed for this article; all quantitative data, including risk estimates and percentages, are cited directly from the original sources to illustrate the strength of the reported physiological associations. Given that consumption is conventionally measured in cups per day, we adopted a standard definition where one cup corresponds to approximately 150 mL of beverage and 100 mg of caffeine; thus, three to four cups equates to a daily intake of 450-600 mL.

Impact of habitual coffee consumption on the cardiovascular system and cardiovascular risk

It has been observed based on a comprehensive dose-response meta-analysis that coffee consumption bears a significant inverse association with both all-cause mortality and cardiovascular disease (CVD) mortality. Specifically, individuals who habitually consume a moderate amount of coffee, typically defined as three to four cups daily, appear to exhibit a substantially reduced risk of death from both all causes and CVD when compared to subjects who infrequently or never consume the beverage. This highlights moderate coffee intake as a potentially favorable protective factor impacting long-term survival [3]. Analysis reports a significant inverse association between coffee consumption and the risk of all-cause mortality, CVD mortality, and coronary heart disease (CHD) mortality among individuals diagnosed with type 2 diabetes mellitus (T2DM). Furthermore, a modest inverse relationship is also established between coffee consumption and the overall risk of experiencing total CVD events and CHD events. In essence, these findings suggest that habitual coffee intake serves as a protective factor that may reduce the mortality burden and cardiovascular event risk within the T2DM patient population [5]. Analysis of the data established a statistically significant inverse association between the level of habitual coffee intake and the frequency of all-cause mortality resulting from heart disease and cerebrovascular disease. Specifically, subjects who reported consuming three to four cups of coffee daily demonstrated a 24% reduction in total mortality when compared to individuals who reported never consuming coffee. These findings collectively suggest that moderate coffee intake may be a favorable lifestyle factor correlated with reduced mortality from major cardiovascular events [4]. Moderate coffee consumption exhibits an inverse association with the risk of developing heart failure (HF). The findings reported by Mostofsky et al. demonstrated a statistically significant J-shaped relationship between coffee intake and HF incidence. This indicates that the risk reduction is not linear. The strongest inverse association, or greatest protective effect, was observed at an intake of four servings per day, which corresponded to an 11% lower risk of HF. However, the protective benefit diminishes and the risk returns to baseline levels when consumption exceeds ten cups of coffee per day. These results suggest that the existing clinical guidelines for HF prevention, which currently imply that coffee intake has harmful effects, may require revision. The data strongly supports the view that coffee consumption, particularly within the moderate range, may actually confer a moderate degree of protection against the incidence of heart failure [13].

Coffee and Atrial Fibrillation

Current evidence does not substantiate the hypothesis that prolonged or long-term exposure to caffeine is associated with an elevated risk of atrial fibrillation (AF). Conversely, data suggest that exposure to lower doses of caffeine may actually confer a minor protective effect against the development of AF [14]. The compound most extensively investigated in coffee, caffeine, is widely hypothesized to significantly influence a neuroendocrine axis. This influence results in elevated serum concentrations of renin, norepinephrine, and epinephrine. Furthermore, at the cellular level, caffeine can delay ventricular myocyte afterdepolarizations by mediating the release of calcium ions from the sarcoplasmic reticulum. Adenosine, a small endogenous molecule primarily generated via ATP catabolism, sees increased release during pathogenic states such as inflammation, hypoxia, and ischemia. Excessive adenosine signaling, whether through elevated release or receptor overexpression within the myocardium, contributes to atrial electrophysiological abnormalities, which ultimately predispose the patient to developing AF. Caffeine is theorized to mitigate this pro-arrhythmic process by functioning as a potent adenosine receptor blocker. Additionally, the anti-inflammatory and antioxidant properties attributed to coffee may offer further protection, preventing the initial genesis of AF [15].

Chemo-preventive potential of coffee constituents in selected malignancies

It is reported that coffee demonstrates a role in various cellular defense mechanisms, including the regulation of DNA repair, phase II enzymatic activity, and apoptosis. Furthermore, coffee components are associated with antiproliferative, antiangiogenic, and antimetastatic effects, collectively suggesting potential protective functions against malignancy progression [6]. Coffee is rich in various bioactive compounds which include phenolic acids and kahweol, both of which possess potent antioxidant and anticarcinogenic activity [16]. Meta-analysis findings demonstrated that coffee consumption was not associated with overall gastric cancer risk. This suggests that, based on the compiled data, habitual coffee intake is considered neutral regarding the overall risk profile for developing gastric malignancy [17]. However, a dose-response analysis regarding coffee consumption and the risk of gastric cancer suggested that there was no significant effect on the cancer risk associated with an increment of one cup per day of coffee intake [16]. High intake of caffeine is positively associated with elevated circulating levels of sex hormone binding globulin (SHBG), and inversely associated with bioavailable testosterone. This modulation of sex hormone bioavailability, specifically the reduction in free testosterone through increased binding by SHBG, is a mechanism that may consequently influence the risks associated with certain hormone-sensitive malignancies, such as endometrial and breast cancers [6]. Coffee may reduce the risk of developing prostate cancer through multiple molecular mechanisms. These include antioxidant and anti-inflammatory activities, provision of protection against DNA damage, modulation of transcriptional factors, regulation via microRNA, and the enhancement of both steroid metabolism and insulin sensitivity (not resistance, as enhanced insulin sensitivity is typically protective against cancer risk) [10]. In the dose-response analysis, the data indicated a reduction in prostate cancer (PCa) risk of approximately 1% for each additional one-cup increment of coffee consumed per day. This suggests a linear protective relationship that scales with daily intake [18]. Coffee's established anti-inflammatory effects contribute to the reduction of tissue damage commonly associated with PCa development. Coffee induces autophagy (cellular self-cleaning), regulates the NF-κB pathway, and reduces the expression of inducible nitric oxide synthase (iNOS). It also lowers the expression of key inflammatory mediators, such as tumor necrosis factor-alpha (TNF-α), interleukin-6 (IL-6), interleukin-8 (IL-8), and C-reactive protein (CRP). Furthermore, coffee actively modulates various transcriptional factors and signaling pathways. Additionally, coffee influences the steroid hormone profile in a manner relevant to PCa risk: it has been shown to increase testosterone and concurrently reduce levels of SHBG, estrogen, and prostate-specific antigen (PSA) [10].

Neuroprotective effects of caffeine in the context of neurodegenerative disorders

Caffeine, the principal psychoactive constituent of coffee, functions as an adenosine receptor antagonist within the central nervous system, thereby counteracting adenosine's sedative effects and promoting wakefulness. This mechanism consistently augments key domains of cognitive performance, including alertness, sustained attention, and processing speed (reaction time) [2]. Complex cognitive functions and mnemonic processes (encompassing working memory, knowledge consolidation, and retrieval) are fundamentally intertwined. Consequently, the neurocognitive benefits associated with coffee consumption demonstrate a clear dose-response relationship, wherein moderate intake levels provide optimal enhancement of these abilities [19]. Coffee presents itself as a compelling pharmacological agent, offering a readily accessible and widely utilized dietary strategy to potentially mitigate age-related cognitive impairment and augment neural resilience (or reserve) [11]. The neuroprotective capacity of caffeine is evidenced by its ability to modulate critical neurotransmitter systems, including the adenosinergic, dopaminergic, and cholinergic pathways, which are centrally implicated in the cellular etiology of neurodegenerative diseases. Furthermore, caffeine consumption has been shown to enhance mitochondrial function, directly promoting neuronal viability, and concurrently regulating specific signaling cascades essential for neuronal protection and survival [20]. Coffee consumption is hypothesized to attenuate neuroinflammation, a key pathological process that is strongly implicated in the onset and progression of several neurodegenerative disorders. These include, but are not limited to, Alzheimer’s disease (AD), Parkinson’s disease (PD), multiple sclerosis (MS), amyotrophic lateral sclerosis (ALS), and Huntington’s disease (HD) [12]. Coffee consumption has been documented as being inversely associated with the risk of neuropsychiatric conditions, specifically indicating a lower incidence of depression [21]. There is biological justification for the theory that coffee consumption could offer a protective effect against the risk of developing AD. Coffee is a highly complex beverage containing various biologically active agents that may exert both beneficial and potentially adverse impacts on the central nervous system. A key hypothesized mechanism for this neuroprotective action centers on adenosine receptor blockade. Caffeine, by acting as an antagonist at these receptors, may lessen the toxic damage induced by β-amyloid, the aberrant peptide known to accumulate in the brains of AD patients [22]. The statistical examination utilizing dose-response analysis established a non-linear and statistically significant correlation between an individual's coffee consumption levels and their subsequent risk of developing dementia. Crucially, this non-linear pattern indicated that the protective benefit was optimized at a moderate intake level. The analysis specifically demonstrated that consuming one to three cups of coffee per day was associated with the most favorable reduction in dementia risk. In clinical terms, the study suggests that more is not necessarily better; the relationship between coffee and dementia risk is governed by a "sweet spot" of moderate consumption [23]. Previous observational investigations suggested that coffee consumption might both reduce the overall risk of PD and postpone the age-at-onset (AAO). However, specific research findings offer a more nuanced interpretation of this protective relationship. Data indicate that although higher coffee intake may successfully delay the emergence of clinical PD symptoms, it does not appear to significantly modify the overall lifetime risk of acquiring the disease nor alter the inherent disease phenotype (the clinical characteristics or trajectory of the illness). In essence, the prevailing conclusion posits that coffee consumption primarily acts by deferring the symptomatic manifestation of PD, rather than reducing the underlying pathological vulnerability or modifying the course of the established illness [24].

Impact of coffee consumption on metabolic homeostasis and endocrine regulation

Coffee stands as a significant contributor to the polyphenol content within the European diet, potentially supplying up to 40% of the total intake of these compounds. The primary class of polyphenols present in coffee is phenolic acids. These compounds have recently garnered attention in epidemiological studies for their promising efficacy against metabolic disorders. Specifically, chlorogenic acids (CGAs), which represent the most abundant phenolic acid group in coffee, have been shown to induce improvements in blood pressure alterations. Furthermore, CGAs possess the capacity to favorably modulate specific metabolic pathways, evidenced by their ability to enhance glucose metabolism and concurrently reduce systemic inflammation and endothelial dysfunction [1]. Studies suggested that both coffee and caffeine intake are significantly inversely associated with the incidence of T2DM. This potential protective correlation appears to be more pronounced in women than in men. It is reported that caffeinated coffee demonstrates a positive relationship with insulin sensitivity, while decaffeinated coffee is favorably related to metrics associated with beta cell function in the pancreas, based on cross-sectional data. Clinical evidence reports that regular coffee ingestion seems to confer beneficial effects concerning subclinical inflammation and high-density lipoprotein cholesterol (HDL-C) levels. Furthermore, when assessed during a two-hour oral glucose tolerance test (OGTT), decaffeinated coffee produced lower glucose levels and a higher insulin sensitivity index compared to the administration of caffeine alone [7]. Observational data indicate that consuming more than three cups of coffee daily is associated with a protective effect against the development of fatty liver disease [25]. It is hypothesized that caffeine may confer protection against the incidence of T2DM through several metabolic actions. These include increasing the metabolic rate and thermogenesis (heat production), stimulating both fat oxidation and the release of free fatty acids from peripheral tissues, and promoting glycogen mobilization within the muscles [7].

Green Coffee Extract Supplementation

Research on green coffee extract (GCE) supplementation has demonstrated notable benefits: a combination of GCE supplementation and resistance exercise is observed to considerably reduce anthropometric parameters (such as body measurements). Furthermore, GCE effectively lowers both systolic blood pressure (SBP) and diastolic blood pressure (DBP). CGA itself contributes to reduced blood pressure and body weight by specifically inhibiting the enzyme 11β-hydroxysteroid dehydrogenase type 1, which is predominantly located in adipose tissues and the liver. This particular enzyme is physiologically involved in converting the hormonally inactive steroid cortisone into the active steroid cortisol. By inhibiting 11β-hydroxysteroid dehydrogenase type 1, CGA effectively reduces active cortisol levels, which consequently lowers blood pressure and promotes weight loss. Supplementation with GCE, at doses ranging from 180 to 376 mg, was observed to induce favorable changes across several key metabolic and anthropometric parameters. Specifically, GCE consumption resulted in significant reductions in waist circumference (an anthropometric measure of visceral adiposity), triglyceride levels, SBP, and DBP. However, it also resulted in a reduction in HDL-C levels. Overall, the data suggest that GCE may be a beneficial intervention for modulating risk factors associated with metabolic syndrome, although the observed decrease in cardioprotective HDL-C warrants further clinical consideration [26].

Impact of coffee consumption on renal function and the risk of nephrolithiasis

Habitual coffee consumption was found to be inversely and dose-dependently correlated with the incidence of chronic kidney disease (CKD), end-stage kidney disease (ESKD), and albuminuria (elevated urinary albumin excretion) [8]. The protective effect of coffee against CKD is primarily attributed to the anti-oxidative and anti-inflammatory properties of its compounds (e.g., caffeine, chlorogenic acid), which mitigate the atherosclerotic burden implicated in CKD pathogenesis. Supporting this, a meta-analysis established a significantly decreased risk of incident CKD among coffee drinkers. Coffee's anti-oxidative effect is the most likely explanation, as atherosclerosis in the kidneys frequently leads to CKD. Caffeine and other compounds like chlorogenic acid are strong antioxidants and anti-inflammatory agents that reduce oxidative stress. Therefore, regular coffee drinkers likely have less inflammation and a lower risk of developing atherosclerosis and endothelial dysfunction [27]. Although coffee intake did not correlate with baseline estimated glomerular filtration rate (eGFR) or the albumin-to-creatinine ratio (ACR) in the overall population, higher consumption was associated with favorable longitudinal eGFR trajectories specifically among high-risk subgroups, such as elderly patients (aged ≥70) and those with obesity [28].

Coffee and Kidney Stones

The inverse association between coffee and caffeine consumption and the incidence of kidney stones is substantiated by multiple pathophysiological mechanisms. Caffeine acts as an adenosine receptor antagonist, exhibiting potent diuretic properties which, when coupled with adequate fluid intake, result in increased urinary flow - a crucial defense against stone formation. Furthermore, caffeine directly impedes calcium oxalate crystal adhesion to the apical surface of renal tubular epithelial cells. An additional protective factor is the inherent richness of coffee plants in citric acid, which raises urinary citrate levels; citrate is a well-established inhibitor of renal calculus formation [29]. Epidemiological data demonstrates that the source of caffeine is crucial; while coffee-derived caffeine is advantageous in mitigating nephrolithiasis risk, caffeine from non-coffee sources is conversely associated with an elevated risk of stone formation. Overall, an inverse association was established between caffeine consumption and the risk of kidney stones. However, subgroup analyses revealed that this beneficial correlation was statistically significant only among white individuals, with the greatest risk reduction observed among women and non-overweight subjects [30].

Hepatoprotective properties of coffee and its impact on the gastrointestinal tract

Caffeine, the principal component of coffee, is hypothesized to provide a direct hepatoprotective effect. Its antifibrotic properties have been demonstrated in in vitro studies through several specific cellular mechanisms. Caffeine mediates the inhibition of focal adhesion kinase, facilitates the induction of filamentous-actin and cAMP expression, and promotes the stimulation of apoptosis specifically within hepatic stellate cells - the key cell type involved in liver fibrosis progression. These mechanisms collectively suggest that caffeine may directly interfere with the cellular signaling responsible for the development of liver scarring [9]. The meta-analysis findings reported by Ebadi et al. confirm the protective role of regular coffee consumption against the risk of developing significant liver fibrosis. Mechanistic evidence from experimental studies indicates that coffee actively mitigates hepatic fibrosis at the cellular level. This is achieved through the down-regulation of profibrogenic genes and by inhibiting both the adhesion and the activation of hepatic stellate cells, which are the primary mediators of liver scarring [31]. Habitual coffee consumption has been strongly implicated in the reduction of risk for developing hepatocellular carcinoma (HCC), the most common form of primary liver cancer. Epidemiological evidence suggests a dose-dependent relationship, wherein higher daily consumption volumes of coffee are associated with a correspondingly greater magnitude of risk reduction for this malignancy. This implies that the chemo-preventive effect of coffee appears to scale positively with increased intake [32]. Bravi F et al. reported significant protective effects of regular coffee consumption on serious liver pathologies in their meta-analysis. They observed a 34% reduction in the risk of primary HCC and a 38% reduction in the risk of chronic liver disease (CLD) when comparing regular coffee consumers to individuals who consumed coffee rarely or never. The authors note that numerous independent studies have previously identified a favorable impact of coffee intake on overall liver function. These collective findings lend strong mechanistic rationale to the observed protective effect against both liver cancer (HCC) and CLD. This beneficial spectrum extends from reducing elevated serum levels of hepatic enzymes, such as glutamyltransferase (GGT) and alanine aminotransferase (ALT) (which serve as key indicators of hepatic distress), to potentially preventing the development of liver cirrhosis, chronic hepatitis B and C infections, and nonalcoholic fatty liver disease (NAFLD) [33]. Findings derived from Wijarnpreecha et al. indicate a significantly reduced risk of developing NAFLD among individuals who habitually consume coffee. Furthermore, their data demonstrated a significantly decreased risk of liver fibrosis among patients already diagnosed with NAFLD who maintained regular coffee consumption. In summation, the authors suggest that coffee intake is associated with a protective effect against both the incidence of NAFLD and the progression to liver fibrosis within the affected patient cohort [9].

Coffee and Cholelithiasis Risk

It is suggested that coffee consumption is associated with a significantly decreased risk of developing gallstone disease, indicating that habitual coffee intake serves as a protective factor that may mitigate the incidence of cholelithiasis [34]. Observational data indicate an inverse association between habitual coffee consumption and the risk of developing both overall and symptomatic gallstone disease. The underlying potential mechanisms that explain this protective correlation are multi-faceted, operating on bile production and gallbladder kinetics: coffee components may stimulate the release of cholecystokinin (CCK), a hormone that enhances gallbladder contraction, contributing to enhanced gallbladder motility while simultaneously inhibiting gallbladder fluid absorption. Furthermore, coffee helps to decrease cholesterol crystallization within the bile. At the enzymatic level, components of coffee may downregulate the hepatic low-density lipoprotein (LDL) receptor and decrease the activity of 3-hydroxyl-3-methylglutaryl CoA reductase (HMG-CoA reductase), both of which influence cholesterol synthesis and regulation. Collectively, these actions promote the efficient flow of less lithogenic (stone-forming) bile, thereby reducing the incidence of gallstone formation [35].

Coffee and Pancreatitis Risk

Heavy coffee consumption is hypothesized to indirectly reduce the likelihood of developing pancreatitis. This potential protective pathway is based on coffee's established association with a lower prevalence of key metabolic risk factors, namely diabetes mellitus and obesity. Given that diabetes mellitus itself constitutes an independent risk factor for acute pancreatitis, and obesity significantly increases the risk of progression to severe acute pancreatitis among affected patients, coffee's favorable impact on these underlying conditions confers a secondary benefit regarding pancreatic inflammation and disease severity [36].

Adverse effects of caffeine consumption

In the context of preventive medicine, moderate coffee intake is considered safe. Maximal mitigation of adverse health outcomes is observed at a daily intake of three to four cups, indicating that the substance possesses substantial prophylactic potential [37]. Supraphysiological caffeine consumption induces acute hemodynamic responses, manifesting as transient hypertension, tachycardia, and palpitations [38]. Coffee-induced lipid elevation is strictly dependent on the brewing method and diterpene dose. While unfiltered preparations (e.g., boiled, French press) contain higher diterpene concentrations associated with hypercholesterolemia, filtered coffee has a negligible impact. Furthermore, the diterpene threshold required to raise cholesterol levels likely exceeds the dose needed for anticarcinogenic benefits [37].

Coffee Intake and Pregnancy Risks

It has been observed that the consumption of both caffeine and coffee correlates with an elevated risk of pregnancy loss. This association exhibits a linear dose-response relationship, indicating that the probability of adverse obstetric outcomes increases proportionally with the level of intake. These findings suggest that prenatal exposure to such substances may act as a precipitating factor for spontaneous abortion [39].

Discussion

This review synthesizes compelling evidence confirming that habitual coffee consumption represents a significant, multifaceted protective factor for the adult population. The findings demonstrate a clear consistency across multiple health domains, where moderate intake contributes substantially to metabolic homeostasis and cardiovascular health, driven by the synergistic action of polyphenols and antioxidants. Specifically, data indicate a robust reduction in all-cause mortality and cardiovascular events, with a 24% reduction in total mortality observed at three to four cups daily [3,4], and an inverse association with heart failure and type 2 diabetes mellitus, challenging previous concerns regarding arrhythmias [7,13,14]. Beyond systemic benefits, coffee constituents exert potent organ-specific effects, significantly reducing the risk of liver fibrosis and hepatocellular carcinoma by 34% [9,32], while simultaneously offering neuroprotection against dementia and neurodegenerative disorders through adenosine receptor blockade [11,23]. Collectively, these data highlight that maintaining a moderate daily coffee intake is critically linked to beneficial outcomes, positioning this beverage as a primary, modifiable dietary component requiring updated public health recognition, with the full scope of this dose-response relationship detailed in Table 1. However, it should be noted that the safety profile of caffeine is strictly dependent on the dose and the method of preparation. While moderate consumption demonstrates prophylactic potential, supraphysiological doses may induce acute hemodynamic reactions, including transient hypertension and tachycardia [37,38]. The brewing method is also a significant factor; in contrast to filtered coffee, unfiltered brews contain higher concentrations of diterpenes, which is associated with a risk of hypercholesterolemia. Furthermore, particular caution is advised during pregnancy, where a linear dose-response relationship between caffeine intake and the risk of pregnancy loss has been demonstrated, suggesting the necessity of limiting exposure during this period [39].

**Table 1:** Summary of Key Health Outcomes and Dose-Response Relationships Associated With Habitual Coffee Consumption CVD: cardiovascular disease, NAFLD: nonalcoholic fatty liver disease

Health domain	Key clinical findings	Optimal intake / risk reduction	References
Cardiovascular system	Significant inverse association with all-cause and CVD mortality; 11% lower risk of heart failure; no elevated risk of atrial fibrillation	3–4 cups/day (24% mortality reduction); J-shaped curve for heart failure	[3,4,13,14]
Metabolic & endocrine	Reduced incidence of type 2 diabetes mellitus (T2DM); improved insulin sensitivity; reduction in waist circumference and blood pressure (via green coffee extract)	Inverse association; protective effect seen at >3 cups/day	[1,7,26]
Hepatology	34% reduction in hepatocellular carcinoma (HCC) risk; 38% reduction in chronic liver disease risk; antifibrotic effects in NAFLD	Dose-dependent (higher intake scales with protection)	[9,31-33]
Neurology	Neuroprotection against Alzheimer’s and Parkinson’s disease; reduced risk of dementia; lower incidence of depression	1–3 cups/day ("sweet spot" for dementia risk)	[19,21,23,24]
Renal function	Inverse correlation with chronic kidney disease (CKD) and kidney stones (nephrolithiasis); diuretic and antioxidant effects	Dose-dependent protective correlation	[8,27,30]
Oncology	Chemo-prevention in specific cancers (prostate, endometrial); neutral effect on gastric cancer	Linear relationship for prostate cancer (approx. 1% reduction per cup)	[6,16,18]

## Conclusions

The comprehensive analysis presented in this review challenges the historical view of coffee as solely a stimulant, positioning it instead as a complex source of bioactive compounds, including chlorogenic acids and polyphenols, that exert significant pleiotropic protective effects. The evidence demonstrates a robust inverse association between moderate coffee consumption (typically three to four cups daily) and all-cause mortality, cardiovascular disease, and heart failure, often following a "J-shaped" dose-response curve. Beyond cardiovascular health, habitual intake exerts profound hepatoprotective actions, reducing risks of fibrosis and hepatocellular carcinoma, while also improving metabolic homeostasis through a lower incidence of type 2 diabetes mellitus and offering neuroprotective benefits against neurodegenerative disorders like Alzheimer’s and Parkinson’s diseases.

However, it is crucial to note that this favorable safety profile is strictly dependent on dose and preparation method. Supraphysiological doses may induce acute hemodynamic reactions, unfiltered brews are associated with hypercholesterolemia, and specific caution is required during pregnancy due to the dose-dependent risk of pregnancy loss. Consequently, while habitual coffee consumption represents a modifiable lifestyle factor with a highly favorable risk-benefit profile for the general adult population, clinical guidelines should be revisited to acknowledge it as a complementary dietary behavior that supports longevity only when consumed in moderation and with respect to individual contraindications. Future research should further elucidate the molecular mechanisms to refine precision nutrition recommendations, ensuring that the chemo-preventive potential of this beverage is fully but safely utilized in preventive medicine.
